# Deregulation of histone lysine methyltransferases contributes to oncogenic transformation of human bronchoepithelial cells

**DOI:** 10.1186/1475-2867-8-15

**Published:** 2008-11-03

**Authors:** Hideo Watanabe, Kenzo Soejima, Hiroyuki Yasuda, Ichiro Kawada, Ichiro Nakachi, Satoshi Yoda, Katsuhiko Naoki, Akitoshi Ishizaka

**Affiliations:** 1 Department of Pulmonary Medicine, School of Medicine, Keio University, 35 Shinanomachi, Shinjuku-ku, Tokyo 160-8582, Japan; 2 Department of Respiratory Medicine, Yokohama Municipal Citizen's Hospital, 56 Okazawa-cho. Hodogaya-ku, Yokohama, Kanagawa 240-8555, Japan

## Abstract

**Background:**

Alterations in the processing of the genetic information in carcinogenesis result from stable genetic mutations or epigenetic modifications. It is becoming clear that nucleosomal histones are central to proper gene expression and that aberrant DNA methylation of genes and histone methylation plays important roles in tumor progression. To date, several histone lysine methyltransferases (HKMTs) have been identified and histone lysine methylation is now considered to be a critical regulator of transcription. However, still relatively little is known about the role of HKMTs in tumorigenesis.

**Results:**

We observed differential HKMT expression in a lung cancer model in which normal human bronchial epithelial (NHBE) cells expressing telomerase, SV40 large T antigen, and Ras were immortal, formed colonies in soft agar, and expressed specific HKMTs for H3 lysine 9 and 27 residues but not for H3 lysine 4 residue. Modifications in the H3 tails affect the binding of proteins to the histone tails and regulate protein function and the position of lysine methylation marks a gene to be either activated or repressed. In the present study, suppression by siRNA of HKMTs (EZH2, G9A, SETDB1 and SUV39H1) that are over-expressed in immortalized and transformed cells lead to reduced cell proliferation and much less anchorage-independent colony growth. We also found that the suppression of H3-K9, G9A and SUV39H1 induced apoptosis and the suppression of H3-K27, EZH2 caused G1 arrest.

**Conclusion:**

Our results indicate the potential of these HKMTs in addition to the other targets for epigenetics such as DNMTs and HDACs to be interesting therapeutic targets.

## Background

Alterations in the processing of the genetic information in carcinogenesis result from stable genetic mutations involving tumor suppressor genes, oncogenes and DNA stability genes as well as from potentially reversible epigenetic changes leading to modifications in gene function [1,2]. It is well established that epigenetic modifications of nucleosomal histones are central to proper gene expression and aberrant DNA methylation of genes play an important role in tumor progression. However, still relatively little is known about histone modifications, especially methylation, with respect to tumorigenesis. The N-terminus of histone tails is modified by amino-acid phosphorylation, acetylation or methylation to form a code for specifying downstream events and consequently a certain chromatin structure. Tens of histone lysine methyltransferases (HKMTs) have been identified and histone lysine methylation is now considered to be a critical regulator of transcription [3,4].

Histone lysine methylation may have positive or negative effects on transcription depending on the methylation sites and also methylation status [5,6]. To date, there are five lysines within histone H3 (K4, K9, K27, K36 and K79) that have been shown to be methylated by specific HKMTs, such as SET9 (for K4), SMYD3 (K4), SUV39H (K9), SETDB1 (K9), G9A (K9), Ezh2 (K27) and DOT1L (K79) [7-13]. Recent advances has revealed that tri-methylations of H3-K27, H3-K9 and H3-K79 are associated with gene repression, whereas mono-methylations of H3-K27, H3-K9 and H3-K79 as well as methylations of H3-K4 are associated with activation [6]. Repressive histone lysine methylation sites at H3-K9 and H3-K27 have been detected at the promoter regions of aberrantly silenced tumor suppressor genes in cancer cells, together with increased DNA methylation and reduced amounts of activating chromatin modifications such as histone acetylation [14,15]. Therefore, HDACs and DNMTs already have emerged as prominent drug targets in epigenetic cancer therapy. Currently, it is apparent that certain histone lysine methylation, along with DNA methylation, establishes the framework for long-term epigenetic maintenance since recent studies have revealed a complex process that controls aspects of short- and long-term transcriptional regulation, in addition to the propagation of bulk chromosome structure and stability [16].

In the present study, we observed high expression levels of several HKMTs in non-small cell lung cancer (NSCLC) cell lines. To elucidate the involvement of histone lysine methyltransferases in tumorigenesis and to determine the potential of these HKMTs as therapeutic targets, we evaluated the effect of suppressing these HKMTs on cell proliferation and tumorigenesis in a Ras-transformed model of human bronchoepithelial cells. Based on an approach established by others, we sequentially introduced human telomerase (hT), SV40 large T antigen (LT) and activated Ras into NHBE cells [17]. Introduction of hT and LT into NHBE cells had immortalized them and additional introduction of Ras had fully transformed them assessed by colony formation in soft agar [18]. Then, we evaluated the effect of siRNA mediated gene suppression of each HKMT on cell proliferation and tumorigenesis in Ras-transformed model human bronchoepithelial cells.

## Results

### Differential Gene Expression of H3-KMTs in immortalized LT-hT-NHBE cells, transformed Ras- LT -hT-NHBE cells and NSCLC cell lines

**Figure 1:**
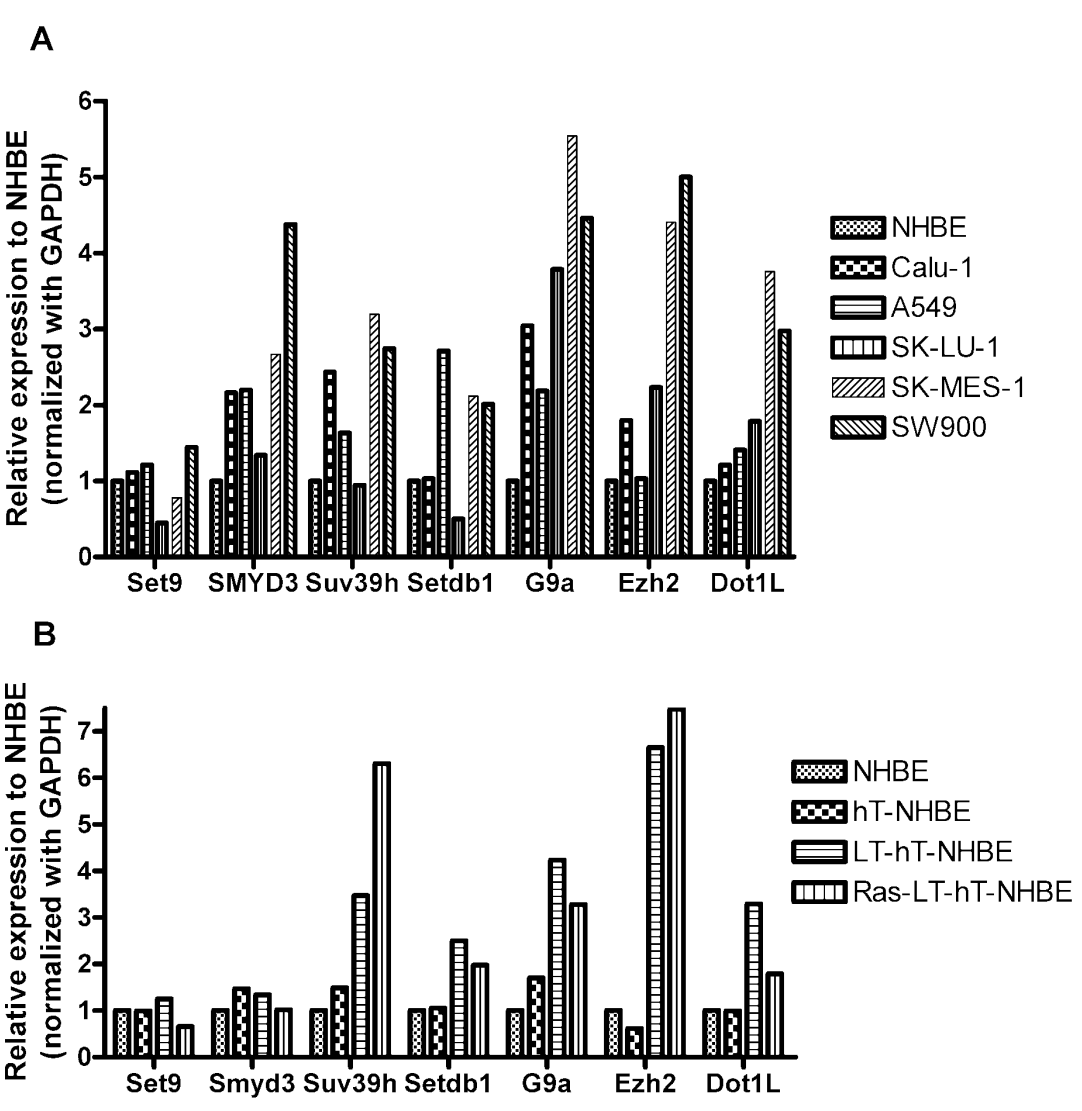
**Expression of Repressive Histone Lysine Methyltransferases (H3K9-MTs and H3K27-MTs) is increased in lung cancer cell lines and after the immortalization and transformation in human bronchoepithelial cells.** A. Quantitative RT-PCR shows mRNA expression of HKMTs are overexpressed in non-small cell lung cancer cell lines A549, Calu-1, SK-LU-1, SK-MES-1 and SW900 except for H3-K4 MT SET9 which remained at the same level compared to NHBE cells. Human glyceraldehyde-3-phosphate dehydrogenase (GAPDH) was used for normalization of cDNA input. Fold differences of mRNA expression compared with NHBE cells (arbitrarily assigned a value of 1). B. Quantitative RT-PCR analysis shows increased mRNA of SUV39H, SETDB1, G9A, EZH2 and DOT1L after immortalization by introduction of hTERT (hT) and SV40 large T antigen (LT) and transformation by introduction of activated H-Ras (Ras) compared with parental Normal Human Bronchoepithelial (NHBE) cells while transcription activating H3-K4 MTs (SET9 and SMYD3) mRNA expression stayed at a similar range compared to NHBE cells. Columns, average from duplicate wells.

First, we examined the expression levels of HKMTs in 5 non-small cell lung cancer cell lines, namely A549, Calu-1, SK-LU-1, SK-MES-1 and SW900. Most HKMTs were up-regulated in the majority of NSCLC cell lines compared to NHBE cells, while only SET9 remained at the same level as in NHBE cells (Fig. 1a). We next found that the expression levels of H3-K4 MTs, SET9 and SMYD3 remained at similar ranges after introduction of LT and activated H-Ras into NHBE cells, while all of the other HKMTs, i.e.; H3-K9, H3-K27, H3-K79 HKMTs, increased after immortalization and transformation (Fig. 1b).

### Suppression of H3-K9 or H3-K27 HKMT resulted in reduced cell proliferation in immortalized and transformed NHBE cells

**Figure 2:**
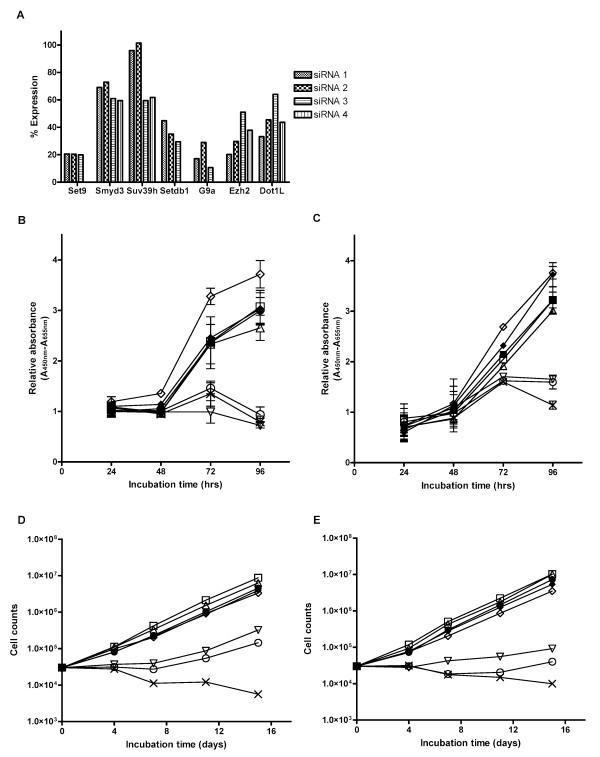
**Suppression of H3-K9 or H3-K27 HKMT resulted in reduced cell proliferation in immortalized and transformed NHBE cells.** A. Quantitative RT-PCR shows different level of knock-down efficiency for siRNAs designed specifically to each target HKMT gene in Ras transformed human bronchoepithelial cells at 48 hours after the transfection of siRNAs. Human glyceraldehyde-3-phosphate dehydrogenase (GAPDH) was used for normalization of cDNA input. Relative expression compared to siRandom non-targeting control treated Ras-LT-hT-NHBE transformed cells. Columns, averages from duplicate wells.B.C. Colorimetric cell enumeration assay shows significant reduction of cell growth after 72 hours of the siRNA treatment of EZH2, G9A and SUV39H compared to the random non-targeting control siRNA in both immortalized (B) and Ras transformed bronchoepithelial cells (C) while interference of the other HKMTs did not affect cell growth rate in these cells. Points, averages from duplicate plates; bars, SD. D.E. Direct cell number counts after a longer incubation period shows significant reduction of cell growth with suppression of EZH2, G9A and SUV39H compared to the siRandom control in both immortalized (D) and transformed cells (E), consistent with colorimetric assay for a shorter incubation period. After a longer period of incubation, inhibitory effect of growth became more prominent in the cells interfered with EZH2 expression than cells suppressed with G9A or SUV39H expression. Meanwhile, siRNA treatment of SET9, SMYD3, SETDB1 and DOT1L did not alter the cell growth rate. (■, black filled square), siRandom, (□ empty square), siSET9, (△ empty triangle pointing up), siSMYD3, (◇ open diamond), siSETDB1, (▽ empty triangle pointing down), siSUV39H, (○ open circle), siG9A, (× cross), siEZH2, (◆ filled diamond), siDOT1L.

To evaluate the function of these HKMTs in the immortalized and the transformed cells, we treated transformed NHBE cells with siRNAs for 7 HKMTs, namely SET9, SMYD3, SETDB1, SUV39H, EZH2, G9A and DOT1L (Fig. 2). The level of each gene mRNA expression was evaluated by quantitative RT-PCR at 48 hours after transient transfection of siRNA (Fig. 2A). Among the 3 or 4 siRNAs tested for each HKMT, the most efficient siRNA sequence was selected for each gene for further experiments. We then evaluated cell proliferation using a colorimetric assay for immortalized cells and transformed cells treated with each siRNA. The cell proliferation was significantly reduced in both immortalized and transformed NHBE cells after 72 hours for the siRNA treatment of EZH2, G9A and SUV39H compared to the random non-targeting control oligonucleotides treatment, while H3-K4 or H3-K79 knock down did not change the cell proliferation levels (Fig. 2B and 2C). We also prepared cell growth curve for immortalized and transformed NHBE cells treated with siRNA. During the cultivation period, as we observed the recovery of the mRNA expression levels had increased by up to about 70% of the untreated level at day 7 (data not shown), we performed a second transient siRNA transfection on day 8 to observe the cell growth only. The cell growth rates for both immortalized and transformed NHBE cells were significantly slowed by siRNA treatment of EZH2, G9A and SUV39H compared to the control oligonucleotide treatment, however, siRNA treatment of SET9, SMYD3, SETDB1 and DOT1L did not alter the cell growth rate (Fig. 2D and 2E). The reduction of cell growth was more notable when a H3-K27 MT, EZH2, was knocked down compared to when any other H3-K9 MTs was suppressed.

### Suppression of H3-K9 or H3-K27MT attenuated anchorage independent cell growth ability of transformed NHBE cells

**Figure 3:**
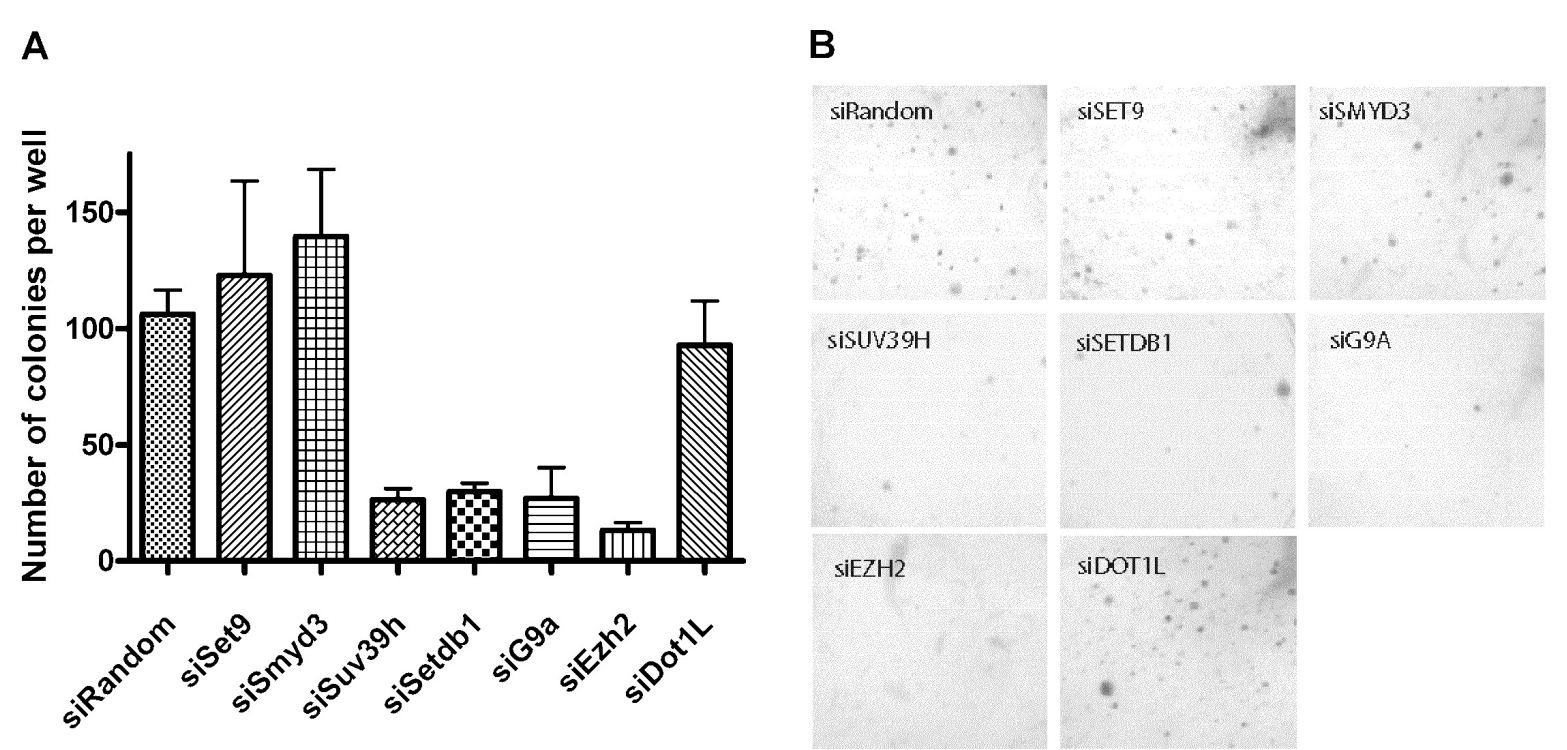
**Suppression of H3-K9 or H3-K27MT attenuated anchorage independent cell growth ability of transformed NHBE cells.** A. Anchorage independent growth on soft agar is inhibited in Ras transformed human bronchoepithelial cells knocked down with all H3K9 MTs, SETDB1, G9A and SUV39H or H3K27 MT, EZH2 compared with the cells transfected with siRandom non-targeting control, indicating that SETDB1 is only required for anchorage independent growth as it did not affect general cell growth while other H3K9 MTs are required for cell growth. Suppression of transcription activating H3KMTs; SET9, SMYD3 or DOT1L did not reduce colony formation in soft agar. The experiments are done in triplicates and repeated twice. Columns, averages; bars, SEM. B. Photographs of representative wells.

Since the expression levels of H3-K9 MTs and H3-K27 MT were almost identical between the immortalized and the transformed NHBE cells, the roles of these HKMTs for transformation induced by activated H-Ras were not clear. To determine if the H3-K9 and H3-K27 MTs play roles in transformation after immortalization, we evaluated the ability of anchorage independent cell growth of transformed NHBE cells treated with siRNA for each HKMT. Compared to control siRNA treated transformed NHBE cells, The cells knocked down with siRNAs for either of 3 H3-K9 MTs or H3-K27 MT formed significantly reduced number of colonies in soft agar compared to the cells treated with the random control siRNA, while H3-K4 MTs or H3-K79 MT knocked down cells did not show any change in the number of colonies or the morphology of the cells (Fig. 3A and 3B). Notably, SETDB1 is also essential for transformation, despite no apparent role in immortalization.

### Suppression of EZH2, but not of H3-K9 MTs, induced cell cycle arrest in immortalized and transformed cells

**Figure 4:**
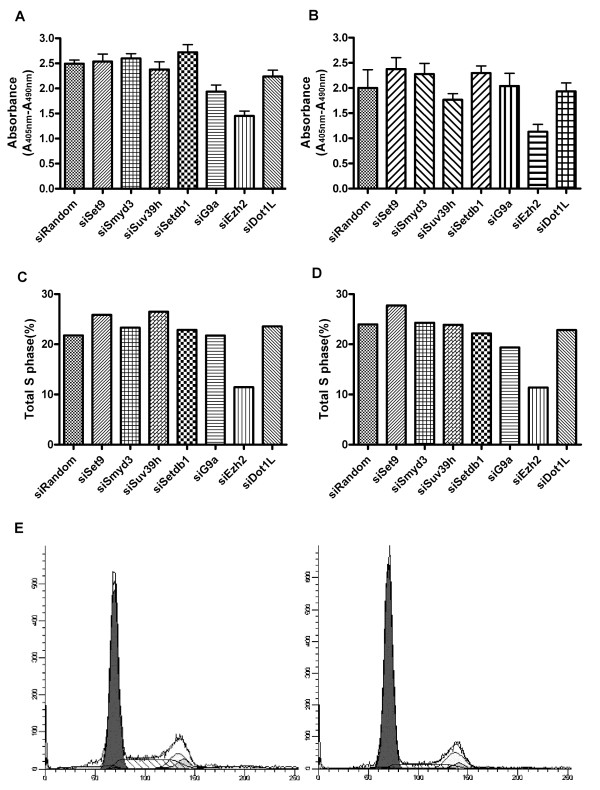
**Suppression of H3K27-MT, EZH2, but not of H3-K9 MTs, induced cell cycle arrest in immortalized and transformed cells.** A.B. BrdU incorporation assay reveals DNA synthesis reduction by interfering with EZH2, a H3K27MT in both immortalized (A) and Ras transformed human bronchoepithelial cells (B). Suppression of H3K9 MTs, SETDB1, G9A or SUV39H did not affect the DNA synthesis level in either of these cells. The experiments are done in triplicates and repeated twice. Columns, averages; bars, SD. C.D. Cell cycle analyses show total S phase reduction only after the treatment of siRNA for H3K27 MT, EZH2 suppression, compared to the cells treated with siRandom non-targeting control both in immortalized (C) and Ras transformed bronchoepithelial cells (D), consistent with the finding on BrdU incorporation assays. None of the other cells interfered with other HKMTs shows total S phase reduction, again consistent with BrdU incorporation assays. (E) Representative cell cycle analysis for the Ras transformed cells treated with siRandom negative control (left panel) and the cells treated with siEZH2 (right panel).

We evaluated the DNA synthesis using a BrdU incorporation assay. DNA synthesis was reduced by suppression of H3-K27 MT, EZH2, in both immortalized and transformed NHBE cells. Meanwhile, suppression of H3-K9 MTs did not affect the DNA synthesis level in these cells (Fig. 4A and 4B). We also evaluated the cell cycle, specifically G1 arrest, by calculating S phase cells based on cellular DNA content in immortalized and transformed cells treated with siRNAs. We found results similar to those of the BrdU incorporation assay, that is, total S phase was only reduced in the cells treated with siRNA for EZH2 (Fig. 4C and 4D).

### Apoptosis was induced by suppression of H3-K9 MTs, G9A or SUV39H

**Figure 5:**
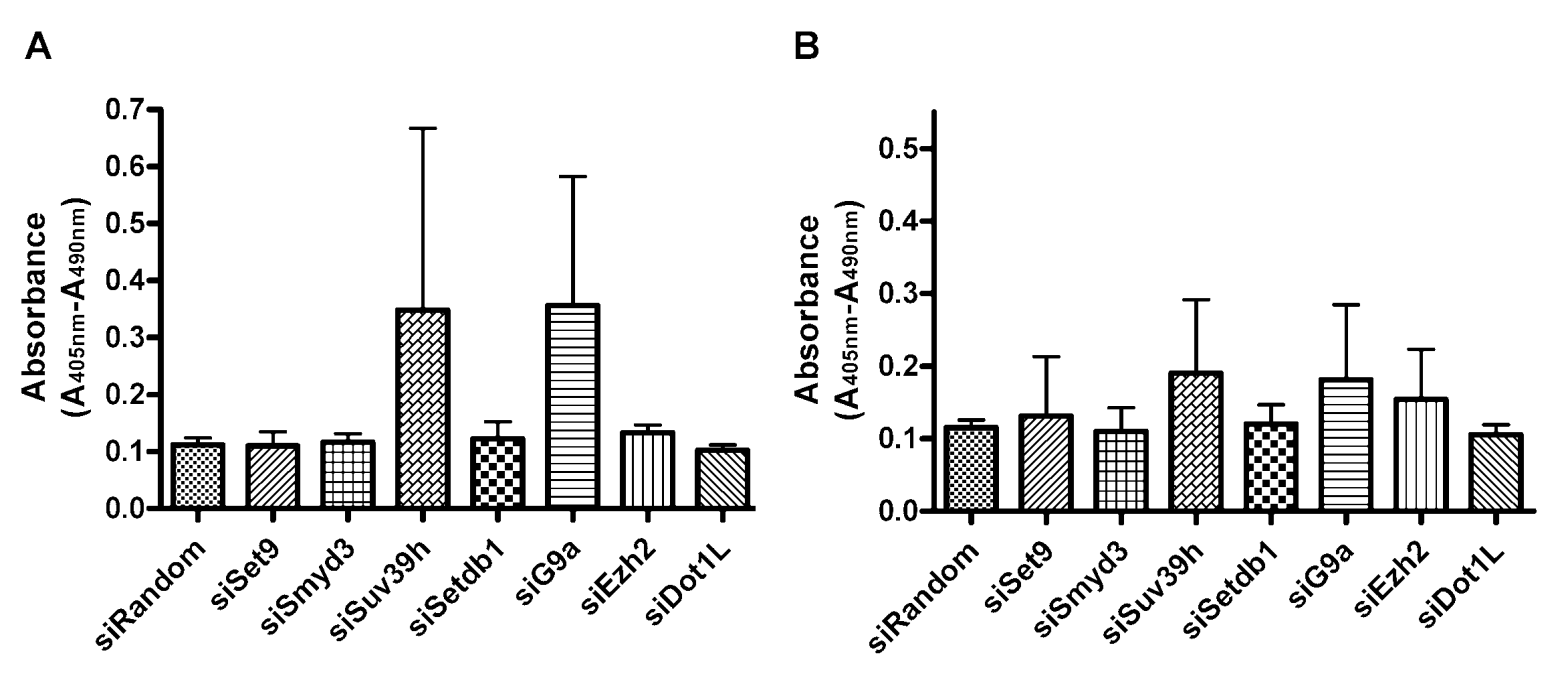
**Apoptosis was induced by suppression of H3-K9 MTs, G9A or SUV39H.** A. ELISA-based apoptosis analysis shows much higher cytoplasmic histone-associated DNA fragments in the immortalized human bronchoepithelial cells knocked down with G9A or SUV39H compared to the cells siRandom non-targeting control although statistical significance is not reached. B. Apoptosis level seemed not be affected by suppressing any HKMTs in Ras transformed bronchoepithelial cells. The experiments are done in triplicates and repeated twice. Columns, averages; bars, SD.

Next, we analyzed apoptosis levels in cells treated with siRNAs to ascertain whether the cell growth inhibition observed was related to apoptosis. Although statistical significance was not reached, apoptosis levels in G9A or SUV39H knocked down cells were much higher than the negative control in immortalized NHBE cells, while apoptosis levels appeared to be unaffected by suppressing any HKMTs in transformed NHBE cells (Fig. 5A and 5B).

## Discussion

Histone methylation is catalyzed by histone methyltransferases, which are a large family of enzymes that have specificity for a histone, the modification site (lysine or arginine), and chromatin region [19]. The identification of SUV39H1 and the finding that Heterochromatin Protein 1 (HP1) binds specifically to methylated H3-K9 revealed a critical role for H3-K9 methylation in heterochromatin formation and epigenetic control of transcription [9,20-24]. HP1 can associate with many other proteins, including HDACs, and RNAs to suppress the expression of certain genes [25,26]. In addition to SUV39H, two other HKMTs, G9A and SETDB1, have HKMT activity toward H3-K9 [10,11]. There has been only one major HKMT shown to have activity specific to H3-K27. EZH2 is a SET domain containing HKMT and has been established as a component of the minimum functional core complex called polycomb repressive complex 2(PRC2) with SUZ12 and embryonic ectoderm development (EED) exerting gene silencing [12,27]. SMYD3 (SET-and MYND-domain containing protein 3) is a H3-K4 MT and sequence-specific DNA binding protein that is over-expressed in other cancers, such as colorectal and hepatocellular carcinomas. SMYD3 is involved in the activation of oncogenes and genes associated with cell-cycle regulation [8]. Dot1L is a non-SET domain containing HKMT specific to H3-K79 [13].

In the present study, we have clearly demonstrated that transcriptionally repressive histone methyltransferases, namely H3-K9 and H3-K27 MTs were increased after sequential introduction of SV40 large T antigen and Ras into NHBE cells whereas the active methyltransferases H3-K4 MTs were not. Moreover, inhibiton of these repressive HKMTs by siRNA lead to growth inhibiton of immortalized as well as transformed human bronchoepithelial cells and also lead to the suppression of anchorage independent cell growth of transformed cells.

Introduction of SV40 large T antigen in NHBE cells renders over-expression of H3-K27 MT, EZH2. It has been shown that deregulation of EZH2 may result in alteration of the chromatin structure and deregulation of the downstream targets of the EED/EZH2 complex [28], and EZH2 is crucial during embryonic development [29]. In breast and prostate cancers, EZH2 over-expression is linked to aggressive tumor formation and poor prognosis [30,31] and in a subset of cancers, the EZH2 locus is amplified resulting in EZH2 over-expression [31,32]. We have demonstrated here that the suppression of EZH2 function leads to growth inhibition of immortalized and transformed bronchoepithelial cells as well as the inhibition of anchorage independent cell growth. We also showed that the suppression of EZH2 resulted in restricted cell cycle, G1 arrest, which is consistent with the previous report [33]. They found that Ezh2 acted down stream of the pRB-E2F pathway and was required for entry into S phase. On the other hand, it has been also shown that EZH2 is transcriptionally regulated by the pRB/E2F pathway [34,35], and activated p53 has also been shown to suppress EZH2 expression [36]. In our model of immortalized LT-hT-NHBE cells and transformed Ras-LT-hT-NHBE cells, both pRB and p53 pathways were inactivated by the introduction of large T antigen [18]. Therefore, the suppression of pRB and p53 also observed in most cancers results in up-regulation of EZH2, leading to deregulation of transcriptional signals and playing a role in maintaining the immortalized phenotype in cancer cells as well as in our cells.

Recent advances in stem cell science have revealed that polycomb group proteins including EZH2 are essential epigenetic gene silencers critical for maintenance of embryonic and adult stem cells [37-39]. This concept is now suggested to be extended to cancer stem cells. Down regulation of EZH2 may cause stem cells to be in a transcriptionally active chromatin state and may cause them to lose the character of stemness. This may be one of the explanations as to why EZH2 inhibition exhibited the strongest effects on cell proliferation and anchorage-independent cell growth. It is possible that inhibition of EZH2 might affect the fate of cancer stem cells, although we were unable to demonstrate the evidence for the existence of stem cells in our experiments.

G9A, SUV39H1 and SETDB1 are also up-regulated in immortalized bronchoepithelial cells and the suppression of G9A or SUV39H1 function reduced cell proliferation and restored apoptosis. While p53 is suppressed through induction of SV40 large T antigen in these cells, it is suggested that suppression of apoptosis induced by H3-K9 HKMTs and probably by H3-K9 methylation is p53 independent. Suppression of pRB and p53 disrupts the properly regulated target gene repressions and may deregulate G9A and SUV39H1 as a result. Recently, Kondo et al. reported that knocked-down PC3 cells both for G9A and SUV39H also showed inhibited cell growth, however, not by induction of apoptosis but by cellular senescence [40]. The difference in the mechanisms for inhibition of cell growth may be due to the cell types used and also due to the techniques used for RNA interference. They established stable clones of PC3 cells, in which either of 2 HKMTs was downregulated by shRNA, whereas we used the cells with transient transfection of siRNAs. We also found that suppressing all 3 H3-K9-HKMTs reduced anchorage independent cell growth. As the suppression of either G9A or SUV39H1 induced apoptosis in transformed NHBE cells, this phenomenon might be explained by the induction of apoptosis. However, as the suppression of SETDB1 was not shown to alter apoptosis levels in these cells, there could be a different mechanism of action to prevent anchorage independent cell growth by a specific SETDB1 function.

As suggested by the normal expression in transformed cells, these results show that suppression of H3-K4 HKMT does not play a role in cell proliferation. H3-K4 tri-methylation is generally associated with a high transcriptional activity [7]. Recently, it was observed that highly tri-methylated H3-K4 was associated with the actively transcribed hTERT gene in telomerase-proficient tumor cells [41]. SMYD3-mediated tri-methylation of H3-K4 functions as a licensing element for subsequent transcription factor binding to the hTERT promoter [42]. The reason why the suppression of SMYD3 did not alter cell proliferation in transformed NHBE cells might be explained by the fact that the hTERT was stably expressed in our hTERT-introduced model.

DOT1L is a non-SET methyltransferase with specificity for H3-K79 and is the only known enzyme with this histone specificity. Although DOT1L contributes to leukemic transformation through H3-K79 methylation [43], and is over-expressed in our immortalized and transformed NHBE cells, Dot1L does not seem to promote the cell proliferation of these cells.

It used to be simply thought that methylation of H3-K9 and H3-K27 was generally associated with repression, whereas methylation of H3-K4, -K36, and -K79 was implicated in the transcriptional activation process [44,45] independent of methylation status, however, it has been revealed that the situation is more complicated. Profiling of histone methylations in the human genome by a comprehensive approach demonstrated that mono-methylations of H3-K27, H3-K9, H4-K20 and H3-K79 as well as mono-, di- and tri-methylations of H3-K4 were linked to gene activation, whereas tri-methylations of H3-K27, H3-K9 and H3-K79 were linked to repression [6]. Tens of genes responsible for histone lysine methylation have been identified and classified so far [4], however, methylation pattern of each H3 lysine site by corresponding HKMT is still controversial. So it will be important to identify specific genes affected by the inhibition of HKMTs and also to investigate methylation status of corresponded lysines of histone H3 by chromatin immunoprecipitation assay to clarify the precise mechanism for growth inhibition in our model system for further experiments.

## Conclusion

In conclusion, we have demonstrated that deregulation of H3-K9 and H3-K27 contributes to oncogenic transformation of human bronchoepithelial cells. Anchorage-independent cell growth was reduced by suppression of any H3-K9 MTs (G9A, SUV39H1 or SETDB1) or H3-K27 MT (EZH2) by inducing apoptosis except for SETDB1 or G1 arrest, respectively, though the precise mechanisms remain to be elucidated. Thus, these HKMTs, either H3-K27 or H3-K9 MTs, may be potential targets for therapeutic drugs to treat lung cancer.

## Methods

### Transformed model of human bronchoepithelial cells

Phoenix producer cells were transfected with the following plasmids using FuGene 6 (Roche Molecular Biochemicals, Indianapolis, IN, USA): pBABE-puro-hTERT and pBABE-hygro-ras-V12 (kind gifts from RA Weinberg, Massachusetts Institute of Technology, Cambridge, MA, USA), and pBABE-puro-U19 and pLXSN-U19 (a kind gift from J DeCaprio, Dana Farber Cancer Institute, Boston, MA, USA). U19 is an SV40 large T (LT) antigen variant lacking the SV40 origin DNA binding motif (Rios, 1990 J Cell Physiol 145:434). Supernatants were used to infect NHBE cells (TaKaRa Bio, Shiga, Japan).

### Cell culture

Five NSCLC cell lines; A549, Calu-1, SK-MES-1, SK-LU-1 and SW-900, were cultured in complete Dulbecco's Modified Eagle Medium supplemented with 10% fetal bovine serum and incubated at 37°C with 5% CO_2_. Immortalized and transformed models of NHBE cells were cultured in Small Airway Growth Medium (SAGM; TaKaRa Bio, Shiga, Japan) at 37°C in 5% CO_2_. (Logarithmically growing cell cultures were used for all experiments described below.)

### Quantitative RT-PCR

Total cellular RNA was prepared from the cells by using an RNeasy Mini Kit (Qiagen, Valencia, CA, USA) and 0.5 mg of the RNA was then reverse transcribed to cDNA using an Omniscript RT kit (Qiagen). For quantitative RT-PCR analysis, the cDNA was combined with gene-specific forward and reverse primers for each HKMT and a SYBR Green PCR master mix and subjected to real time fluorescence detection PCR using an ABI Prism 7000 Sequence Detection System (Applied Biosystems, Foster City, CA, USA). Human glyceraldehyde-3-phosphate dehydrogenase (GAPDH) was used for normalization of cDNA input. The thermal cycling conditions were as follows: initial denaturation at 95°C for 15 min, 45 cycles of 95°C for 15 s, 58°C for 15 s and 72°C for 30 s, followed by melting temperature analysis (72–99°C with 1°C increments). The amplification was specific as judged by melting temperature analysis and agarose gel analysis. The experiments were performed in duplicate and twice. The following oligonucleotides were used as primers.

SET9 forward primer: TTCACTCCAAACTGCATCTACGA

SET9 reverse primer: GGGTGCGGATGCATTTG

SMYD3 forward primer: CCCAACTGTTCGATTGTGTTCA

SMYD3 reverse primer: TCCTCTCCCACCTCGATGTC

SUV39H forward primer: CTGCCCATCTACGAGTGCAA

SUV39H reverse primer: TACCCTTCTGTACCACACGATTTG

SETDB1 forward primer: GACTCTCTGAGACAACTTCCAAGGA

SETDB1 reverse primer: CAGGGATTGAGGGAGGAACA

G9A forward primer: CTCCGCTGATTTTCGAGTGTAA

G9A reverse primer: CTCTGTACGACCCGGTTCTTG

EZH2 forward primer: CAAGCAGTGCCCGTGCTA

EZH2 reverse primer: AGCGGCTCCACAAGTAAGACA

DOT1L forward primer: CATCCGATGGGTCTGTGA

DOT1L reverse primer: TGGTGTCATAGTCAATTAAAACGTAATTC

### Gene knockdown by siRNA

For siRNA-mediated down-regulation of HKMTs, each target specific sequence was designed using siDirect software provided by RNAi Co., Ltd (Tokyo, Japan) and synthesized by Proligo Japan KK (Kyoto, Japan). Three or four target sequences were designed for each HMT gene and transient transfection of siRNAs was performed. All siRNA experiments were conducted at a final concentration of 20 nmol/L duplex siRNA. For transfection into the cells, Lipofectamine RNAiMAX (Invitrogen, Carlsbad, CA, USA) was used according to the manufacturer's protocol. Knockdown of each HKMT expression was confirmed using quantitative reverse transcription-PCR. Non-targeting control siRNA was provided by RNAi Co., Ltd as well. The following were used as siRNA targeting sequences that were most effective for each gene:

Non-targeting control; GUACCGCACGUCAUUCGUAUC

SET9; GGGAGUUUACACUUACGAAGA

SMYD3; CCGCGUCGCCAAAUACUGUAG

SUV39H; GAAAUGGCGUGGAUAUCCAGA

SETDB1; CAGCCCGGCGUCGAGUUAACC

G9A; CAUGACUGCGUGCUGUUAUUC

EZH2; GAAUGCCCUUGGUCAAUAUAA

DOT1L; GGCGAGCCAGUCAAUAGCAGC

### Cell enumeration assay and cell growth rate analysis

Cell Proliferation Reagent WST-1 (Roche, Indianapolis, IN, USA) was used for the colorimetric cell enumeration assay according to the manufacturer's protocol. One day before siRNA transfection, 1 × 10^4 ^cells were plated in 100 μl of the growth medium for each 96-well culture plate. Following the siRNA transfection, cells were incubated for 24, 48, 72 and 96 hours at 37°C in a 5% CO_2 _environment. WST-1 reagent was then added to each well, cells were incubated for an additional 4 hours, and then the absorbance of the samples was measured at a wavelength of 450 nm against a background control using a microplate reader (Bio-Rad Laboratories, Hercules, CA, USA). For cell growth rate analysis, one day before siRNA transfection, 3 × 10^5 ^cells were plated in growth medium in 12-well culture wells and then the cells were collected by trypsinization 48 hours, and 7, 10 and 14 days after the siRNA treatment and counted using a cell counter, Z1™ Series COULTER COUNTER^® ^(Beckman Coulter, Fullerton, CA, USA).

### Soft agar colony formation assay

Three ml of 0.6% agarose containing SAGM per well was prepared in a standard 6-well cell culture plate. Then, 1.5 ml of 0.3% agarose containing SAGM with a suspension of 0.5 × 10^4 ^cells treated with siRNAs was added on top of the basal 0.6% agarose containing medium. After 14 days of incubation at 37°C in 5% CO_2_, colony growth in soft agar was assayed by visually counting the colonies. A colony was defined as >100 cells in one site. Experiments were performed in triplicate with two independent experiments.

### BrdU incorporation assay

Cell Proliferation ELISA BrdU (Roche, Indianapolis, IN, USA) was used for the BrdU incorporation assay. Cells (1 × 10^4^) were plated in 100 μL of SAGM in 96-well culture plates and cultured for 48 hours after the treatment at 37°C in a 5% CO_2 _incubator. The newly synthesized DNAs were labeled with BrdU for an additional 4 hours of incubation, and then the cells were fixed and the DNA was denatured. After incubation with anti-BrdU-POD for 90 minutes, a POD substrate (tetramethyl-benzidine) was added to develop a color to be quantified by measuring the absorbance at the 370 nm with a microplate reader (Bio-Rad Laboratories, Hercules, CA, USA).

### Cell cycle analysis

For starvation, 1.5 × 10^6 ^cells were incubated for 24 hours in 1.0 ml of SAGM without growth supplements for each 6-well cell culture plate at 37°C in a 5% CO_2 _incubator before treatment with siRNAs. Forty-eight hours after the siRNA transfection, the cells were trypsinized and resuspended in 0.5 ml of phosphate Buffered Saline (PBS) and then fixed with 4.5 ml of 70% ethanol for 2 hours on ice. After one wash with PBS, for DNA staining the fixed cells were incubated with DNA fluorochrome, propidium iodine (PI) along with 0.1% Triton X-100 and ribonuclease A for over 30 min at room temperature. Cell fluorescence was measured by a flow cytometry, FACS Flow (BD Biosciences, San Jose, CA, USA). Deconvolutions of the DNA content frequency histograms were analyzed using CellQUEST software (BD Biosciences).

### Apoptosis assay

Apoptosis assay was performed using Cell Death Detection ELISA PLUS according to the manufacturers' protocol (Roche, Indianapolis, IN, USA). Cells (1 × 10^4^) were plated in 100 μl of SAGM in each 96-well culture platse and cultured for 48 hours after siRNA treatment at 37°C in a 5% CO_2 _incubator. After cell lysis, intact nuclei were pelleted by centrifugation and then placed into a streptavidin-coated well of a microplate. A mixture of anti-histone-biotin and anti-DNA-POD were added and incubated. After unbound antibodies were removed by a wash, POD retained in the immunocomplex was determined by adding substrates to develop a color to be quantified by measuring the absorbance at 370 nm with a microplate reader (Bio-Rad Laboratories, Hercules, CA, USA).

## Competing interests

The authors declare that they have no competing interests.

## Authors' contributions

HW carried out most of the experiments and drafted the manuscript. HY participated in the gene knockdown by siRNA and the colony formation assay. IK and IN participated in the quantitative RT-PCR assay. SY and KN participated in the cell cycle analysis and the apoptosis assay. KS and AI designed the project, supervised the experiments and helped to draft the manuscript. All authors read and approved the manuscript.
